# Occupational therapy synergy between Comprehensive Community Based Rehabilitation Tanzania and Heifer International to reduce poverty

**DOI:** 10.4102/ajod.v2i1.48

**Published:** 2013-07-11

**Authors:** Anne Marie W. Hansen, Albert P. Chaki, Ruth Mlay

**Affiliations:** 1 Duquesne University, Pittsburgh, United States; 2 CCBRT-Moshi, Tanzania

## Abstract

**Background:**

This article describes a partnership between a community-based rehabilitation organisation and a non-governmental organisation (NGO) in Tanzania. The partnership focused on income-generating (IG) activities to tackle the problems of poverty faced by families with a child with a disability (CWD).

**Objectives:**

The aim of this case study was to describe the partnership between Comprehensive Community Based Rehabilitation Tanzania in Moshi (CCBRT-Moshi), a non-governmental organisation, and families to create an income-generating business, namely raising goats.

**Method:**

This was a team approach between CCBRT-Moshi and Heifer International, an organisation that focuses on IG activities to create a synergy or partnership between community-based rehabilitation and IG activities.

**Results:**

This partnership between occupational therapy rehabilitation services at CCBRT-Moshi and the NGO resulted in strengthening the effectiveness of occupational therapy services and leaving a more lasting impact on the people they served within the community by helping to reduce poverty in addition to providing rehabilitation and prevention interventions.

**Conclusion:**

This collaboration was successful as it provided a means for families to generate income from raising goats. Although the results have not been empirically verified, observational and anecdotal evidence suggests that families with CWDs have better quality of life and ultimately improved health through this synergistic partnership.

## Introduction

Poverty and hunger are two major obstacles that occupational therapists face when working in community-based rehabilitation (CBR) programmes providing rehabilitation services and improving the quality of life for members of the community. The Comprehensive Community Based Rehabilitation Tanzania in Moshi (CCBRT-Moshi), like many CBR programmes, is based in communities of people who are hungry and live in poverty. This poses a major obstacle in ensuring effectiveness of its services and sustainable community development because these families, and children in particular, face additional severe deprivations of clean water, health services, sanitation, shelter, education and access to information (UNICEF 2009). Comprehensive Community Based Rehabilitation Tanzania programmes aim to empower people with disability and their families to assert their rights and make a contribution to their own livelihood activities through programmes that emphasise prevention of disability, access to economic empowerment activities, inclusion of children with disabilities (CWD) in mainstream schools and physical accessibility of facilities. This article describes a partnership between CCBRT-Moshi and a non-governmental organisation (NGO) that focuses on income-generating (IG) activities to tackle the problems of poverty and hunger at a local level. The aim of the partnership is to work with the family to create an income-generating business, namely raising goats, to reduce poverty and thus hunger. Poverty and hunger are major causes of disabilities and can lead to secondary disabilities for individuals who are already disabled as a result of poor living conditions, poor access to health care and education opportunities (World Bank 2010). Together, poverty and disability create a vicious cycle. Opportunities for IG activities in poor and hunger-stricken communities are limited. Comprehensive Community Based Rehabilitation Tanzania in Moshi’s main focus has been on preventing impairments and disabilities, treating or curing disabilities, improving the physical condition of patients with disabilities and empowering those with disabilities and HIV to assert their rights and to make a contribution to their own livelihood (CCBRT 2012). Comprehensive Community Based Rehabilitation Tanzania has not traditionally focused on IG activities. This case report describes a team approach between CCBRT-Moshi and Heifer International, an organisation that focuses on IG activities to create a synergy or partnership between CBR and IG activities. Through this partnership, occupational therapy rehabilitation services at CCBRT-Moshi has made a stronger and more lasting impact on the people they serve within the community by helping to reduce poverty and thus hunger in addition to rehabilitation and prevention interventions as mentioned above. This case study describes the first such collaboration between CCBRT staff, the family of a child with a disability, and an NGO that focuses on IG activities.

### Background to the study

Tanzania is one of the 189 nations which endorsed the Millennium Development Goals (MDGs) in September 2000 as part of the internationally agreed-upon development goals at the General Assembly of the United Nations (UN 2008). The Millennium Development Goals were established in September 2000, at the Millennium Summit, the largest gathering of world leaders in history. At this summit and through these goals, the participating nations committed to a new global partnership to reduce extreme poverty and set out a series of time-bound targets. These time-bound targets address extreme poverty in its many forms, including income poverty, hunger, disease, lack of adequate shelter and exclusion, while promoting gender equality, education and environmental sustainability (Millennium Project 2006). The goal of utmost importance in Tanzania is MDG No. 1, to eradicate extreme poverty and hunger, as 57.8% of the Tanzanian population is estimated to live under the poverty line of $1.00 per day (WHO 2011).

Poverty hits children hardest as it impacts their health and nutrition, education, participation, and protection from harm and exploitation, and thus creates an environment that is damaging to children’s development in every way – mental, physical, emotional and spiritual (UNICEF 2012). These complex issues challenge occupational therapists to think creatively about client-centred interventions that address more than just the child’s mental and physical needs. In practice, occupational therapists look at the child (or client of any age) as an occupational being, the contexts in which they live (family, home, school, work) and their meaningful occupations (activities of daily living, play, education, work) (Watson & Duncan 2010). When working with a child with a disability, the occupational therapist must look beyond the child, and explore the home and family context. An occupational therapist might ask, ‘Does this child’s family have the ability to feed, clothe and educate their child?’

The population of Tanzania is 43.2 million (Tanzanian Embassy 2012). More than 80% of the population is rural and 7.8% of the population ages 7 years and above have some type of disability or ‘activity limitation’ (Ruyobya *et al*. 2008). This percentage is higher (8.3%) for people living in rural areas (Ruyobya *et al*. 2008). The life expectancy is 53 years for men and 56 years for women, and more than 44% of Tanzanians are younger than 15 years (WHO 2011). Since its independence in 1961, the government of Tanzania has placed poverty and hunger eradication at the top of its national agenda (UN Commission on Sustainable Development 1997). Since 2005, for example, the Tanzanian economy has been affected by daunting local and global challenges. One of these is a severe drought, which adversely affected crop production, livestock and power generation to proportions never experienced in recent decades. At the global level, the economy was negatively impacted by high oil and food prices and the global financial and economic crises (BEST-AC 2010). One of the proposed government intervention measures was the introduction and implementation of social and economic policies that address the issues of poverty and hunger both at national and individual level for more effective distribution and channelling of national resources to ensure greater access to resources and services for all its citizens. Tanzania developed MKUKUTA (Mpango Wa Kukuza na Kuondoa Umaskini Tanzania) National Strategy for Poverty Reduction in 2005 and is currently implementing MKUKUTA II, which has the following objectives:

- ensuring that good governance and accountability prevails in Tanzania as a base for poverty reduction
- ensuring timely and appropriate justice for all, especially the poor and vulnerable groups
- reducing political and social exclusion and intolerance
- ensuring sound economic management
- ensuring adequate social protection and rights of the vulnerable and needy groups with basic needs and services
- focusing on reducing child labour (BEST-AC 2010).

These strategies are aimed at meeting two major targets to be achieved by the government of Tanzania in eradicating poverty and hunger:

- National Target 1: between 1990 and 2015, reduce by half the proportion of people whose income is less than $1.00 a day
- National Target 2: between 1990 and 2015, reduce by half the proportion of people who suffer from hunger.

Tanzania’s Ministry of Finance and Economic Affairs reports that the country is ‘clearly on track to achieving the MDGs related to primary education, child mortality, gender equality, and access to improved sanitation, but is lagging behind in other MDGs’ (United Nations Developmental Programme [UNDP] 2010). Although the spread of HIV is the single most impoverishing force facing people and households in Tanzania today, if not halted and reversed, it threatens not only the achievement of the targets in the poverty reduction strategies MKUKUTA and Zanzibar Strategy for Growth and Reduction of Poverty (MKUZA) but the MDGs more broadly (UNDP 2010). The country still faces huge challenges:

‘[*E*]conomic growth has been neither broad based nor robust enough to lead to a significant reduction in poverty, and indicators for social progress are less than impressive. As a result, the overall human development remains low, with a human development index of 0.398 in 2010, compared to 0.329 in 1990.’ (UNDP 2010)

### The reality of poverty

Agriculture is the leading sector in the Tanzanian economy, accounting for nearly half of the gross domestic product. Eighty-five per cent of the Tanzanian population is involved in farming; it is estimated that there are 4.8 million smallholder farmers and nearly 3.9 million households keeping livestock (Irish Aid 2008). Over 80% of poor Tanzanians live in the rural areas. Tanzanian agriculture is almost completely rain dependent. In years when rainfall is scant, thousands of Tanzanians require food aid to ensure that they do not go hungry. Farmers depend on water, their good health and being free of diseases or illness, yet drought and disease leave them vulnerable (Irish Aid 2008). Additionally, due to poor governance, climate change and the increased frequency of droughts, agricultural production has not been enough to feed its population. The country has been importing between 4% and 10% of its food and receiving food aid to meet production shortfalls (Irish Aid 2008). Therefore it is important to work with families, particularly families with a child with a disability, who depend on agriculture to make a better living, to find a more reliable source of income.

Although the majority of the population survives on agriculture and subsistence farming, the crops often fail or are of poor nutritional quality. This lack of proper farming knowledge and general poverty has led to poor nutrition amongst many Tanzanian families (eds Baker & Pedersen 1992). Pregnant women do not receive proper information on dietary requirements before, during and after delivery, resulting in low birth weights (WHO 2013a). Research has shown that poor nutrition in pregnant women affects an unborn child’s development and can have adverse consequences on a child’s development (Wu, Imhoff-Kunsch & Webb Girard 2012). Malnutrition also has severe implications for a child’s learning capacity and growth. Lack of knowledge and adequate nutrition are contributing factors in the increasing number of children with disabilities (WHO 2013a).

Tanzania’s National Strategy for Growth and Reduction of Poverty has pinpointed agricultural growth as one of the necessary components to reduce poverty (BEST-AC 2010). ‘Agriculture First’ is now a leading campaign in the reduction of poverty and hunger (BEST-AC 2010) and presents an ideal policy framework within which to locate community-based rehabilitation initiatives such as the one described in this article.

### People with disability in Tanzania

The Tanzania Disability Survey (Ruyobya *et al*. 2008) was conducted by the National Bureau of Statistics (NBS) in collaboration with the Office of the Chief Government Statistician, Zanzibar (OCGS) and the Ministry of Health and Social Welfare. This was the first time Tanzania carried out such a comprehensive survey on people with disabilities. The survey was household based and covered both Tanzania Mainland and Zanzibar. The major objective of the 2008 Tanzania Disability Survey was to determine the prevalence of disability in the country. The survey also intended to determine living conditions amongst people with activity limitations. In the 2008 Tanzania Disability Report, 25% of people with disabilities claimed that the physical environment or attitudes of others make it more difficult for them to take part in three complex activities (i.e. taking care of household activities, day-to-day work or schooling, and taking part in community activities). About 40% of people with disabilities reported having problems with accessibility to public transport.

The accessibility of the physical environment largely determines the extent to which persons with disabilities function in an inclusive manner in their communities (Ruyobya *et al*. 2008). Generally about 8 out of every 10 people with disabilities in Tanzania reported an accessible kitchen, living room and toilet, 9 out of 10 people reported to have access to the bedroom, and 6 out of 10 had access to the dining room (with 33% indicating that they do not have a dining room). People with physical and visual disabilities reported more difficulties with access compared with persons in other categories (Ruyobya *et al*. 2008).

Approximately 1 in every 10 people in Tanzania reported the following as inaccessible: work or school and shops or banks or post office ((Ruyobya *et al*. 2008). About 2 in every 10 people reported their place of worship to be inaccessible. These rates were slightly higher than those reported in regard to the respondents’ homes. This suggests that places outside the home are inaccessible for people with disabilities. In addition, a number of people reported that they ‘never go’ to places outside of their homes. Thirty per cent of all people with disabilities reported that the hospital or clinic was inaccessible. This high inaccessibility rate may be due to physical inaccessibility or lack of services owing to the distance involved (Ruyobya *et al*. 2008).

### Community-based rehabilitation

The World Health Organization (WHO) describes community-based rehabilitation as ‘enhancing the quality of life for people with disabilities and their families, meeting basic needs and ensuring inclusion and participation’ (WHO 2013b). Community-based rehabilitation was initiated in the mid-1980s but has evolved to become a multi-sectoral strategy that empowers persons with disabilities to access and benefit from education, employment, health and social services. Community-based rehabilitation is implemented through the combined efforts of people with disabilities, their families, organisations and communities, relevant government and non-government health, education, vocational, social and other services (WHO 2013b).

Comprehensive Community Based Rehabilitation Tanzania in Moshi is a CBR programme. The staff and administration decided to focus on ensuring better nutrition for mothers and children with a disability so as to help them improve their quality of life and prevent disabilities in unborn children (CCBRT 2012). The CCBRT-Moshi programme is part of the larger CCBRT community programme, with its headquarters 439 kilometres away in Dar es Salaam, the largest city and commercial capital. The Dar es Salaam location serves a population of over two million people. CCBRT is the largest indigenous provider of disability and rehabilitation services in the country, providing quality rehabilitative services to 120 000 people with disabilities and their caregivers each year.

Comprehensive Community Based Rehabilitation Tanzania in Moshi began its work in 1996 and covers 20 wards out of 31 in the Moshi rural district (CCBRT 2012). The centre offers rehabilitation services for children with a disability or health conditions such as hydrocephalus and spina bifida, cerebral palsy, cleft lip and palate, and clubfoot. These and other health conditions create impairments that may lead to activity limitations, especially in rural environments where individuals face participation restrictions associated with physical and attitudinal barriers. The authors of the 2008 Tanzania Disability Survey describe ‘disability’ in accordance with the United Nations Convention on the Rights of Persons with Disabilities:

‘[*P*]ersons with disabilities include those who have long-term physical, mental, intellectual or sensory impairments which in interaction with various barriers may hinder their full and effective participation in society on an equal footing with others.’ (UN 2006)

This definition is in line with how the World Health Organization International Classification of Functioning, Disability and Health (ICF) defines disability as the outcome of the interaction between a person’s health condition and the context in which the person lives (WHO 2013c).

The CBR programme is one of the very few facilities in the Kilimanjaro region providing quality comprehensive CBR services – as established through an external audit confirmed with objective data for children with disabilities (CWDs) and their families in the community. All medical and rehabilitative interventions, including consultations and assessments, non-surgical treatment, therapy, training and special seating clinics, are performed at the rehabilitation centre, The House of Hope, in Moshi.

### Occupational therapy’s unique role

At the heart of the occupational therapy profession is a commitment ‘to advance certain core principles, one of which is the right of all people – including people with disabilities – to develop the capacity and power to construct their own destiny through occupation’ (WFOT 2011). Together with community rehabilitation workers (CRWs), occupational therapists and other health providers, therapists conduct visits to people’s homes and community day-care centres for therapeutic activities and rehabilitation follow-up services. All therapeutic and rehabilitation efforts by occupational therapists in collaboration with other team players are geared towards ensuring that children with disabilities are given an opportunity at full quality of life, and that they grow up to achieve their maximum potential. Occupational therapy’s role in CBR at CCBRT had a particular focus on rehabilitation related to prevention, treatment and maintenance (Watson 2004).

The CCBRT staff found that hunger often jeopardises the success of therapeutic interventions of children with disabilities, such as self-care activities, and school attendance. For example, parents and caregivers often work far from home and do not have the time to follow through on the home exercise programme for their children when they return home from work, as much of their time is spent in meal preparation and caring for other family members. When an occupational therapist makes a follow-up home visit and finds the child alone and the parents and/or caregiver is away, the therapist is not allowed to work with the child due to child protection rights (ILO 2009). Thus, the child does not participate in follow-up exercise or care from the parents or travelling therapist. This is an example of how poverty impacts family life and jeopardises the effectiveness of occupational therapy services. With insight in and commitment to the core principles of occupational therapy mentioned above, an occupational therapist on the CCBRT staff decided that finding ways to reduce hunger and poverty in families of children with disabilities could be crucial to ensuring success in all CBR services. In addition to its focus on rehabilitation in prevention, treatment and maintenance (Watson 2004) this plan enhances CCBRT’s services by focusing on fulfilling one of CCBRT’s main organisational goals – that of supporting children and their families through the development of a plan to support nutrition and economic stability for the entire family.

Due to staff shortages, and more immediate rehabilitation needs of children who come to CCBRT for care, occupational therapists at CCBRT-Moshi have not been trained to provide opportunities for income generating (IG) activities nor has this been a focus of CCBRT. However, CCBRT staff members realised that the work done by occupational therapists and other team members cannot be effective unless the issue of hunger is addressed. With an understanding that context and environment impact one’s daily occupations (eds Kronenberg, Pollard & Sakellariou 2011; eds Watson & Schwartz 2004) the occupational therapists felt they could work with CWDs and their families to develop skills and opportunities for income generation through partnering with organisations that focus on income generation as a poverty-reduction strategy.

### Creating synergy: Pass on the Gift Project

This led to CCBRT-Moshi partnering with Heifer International, an organisation whose mission is to work with communities to end hunger and poverty and care for the earth (Heifer International n.d. b). Recognising the shared mandate and desire to fight poverty and hunger, one of the CCBRT occupational therapists developed a partnership with Heifer International in 2008. Through a partnership between the CCBRT staff, particularly the occupational therapist and Heifer veterinary doctors, they developed a synergy to work together with families of children with disabilities to develop the ‘capacity and power to construct their own destiny through occupation’ (WFOT 2012) by creating an income generating opportunity for the families.

The occupational therapist and CRWs created a survey to identify 100 most poverty stricken families with CWDs to be the first beneficiaries of Heifer International’s Pass on the Gift Project for orphans and vulnerable children (Heifer International n.d. a). Heifer International, through its veterinarians, provides education on the rearing of milk goats while other Heifer specialists teach families how to generate income from the goats. The organisation also provides iron sheets and nails to the beneficiary families and helps with the construction of goats’ huts.

The 100 families were divided into 4 groups of 25 families each, grouping neighbours or families living in close proximity to one another. Each group then elected a leader as the key point of contact for Heifer and CCBRT. Each leader received training, which he or she passed on to the rest of the group, and was responsible for monitoring and reporting the progress of each family’s project. To ensure that the families owned the project, each family was asked to contribute building materials such as sand, stones and timber for completion of the huts.

### Working together to create synergy

Occupational therapists, CRWs, veterinarians and local government leaders work together to supervise and ensure the smooth running of the project activities. Each entity in the partnership shares a unique and an important role, working together to create a synergy that supports each other and the families in ensuring successful and timely construction of the goat huts and other aspects of the project. Under the supervision of the occupational therapist, the CRWs design activities of daily living for children with a disability and teach parents how to guide their children in developing their skills in keeping and caring for their goat at home. These activities include cutting grass for their goat, grooming the goat, leading it to pasture, and providing water and food for the goat. Even if a child is not able to fully participate in these activities, the CRW under the direction of the occupational therapist adapts the environment and the activity in a way that the child can be integrated into an activity. For example, a child in a wheelchair can sit outside the house and keep watch on the goat, rather than sitting alone inside the house.

The role of the veterinarian is to provide health services and appropriate knowledge to parents and children about how to take care of the goats in order to keep them healthy and strong. For example, the veterinarian explains how the goats need exercise every day, proper feeding techniques and how to prevent bloating. Local government leaders and village health workers are also active members of this team. A government leader’s role is to identify new families within the community who have a child with a disability, to introduce them to CCBRT staff and to encourage families to become involved. Village health workers, under the guidance of a CRW, function on the village level and assist the CRW. Village health workers are trained to recognise a disability, guide the community rehabilitation committee on steps to help children with a disability attend school, adults with a disability obtain work and all people with a disability participate in community activities. Whenever possible, their work is carried out in collaboration with representatives from education and social services.

The occupational therapist understands the needs of the child and the family related to disability, including rehabilitation, social inclusion and participation. The occupational therapist assists the family in adapting their home and community environment to be functionally friendly for a child with a disability, including identifying key activities in which the child with a disability can participate, and helps them build their performance skills related to taking care of the goat, in addition to providing disability-related information. Through these numerous activities, the occupational therapist is a unifying presence in the partnership. This creates a unique synergy between disability and poverty reduction by passing on to the families the gift of education and IG activities for poverty reduction.

The family representative serves as the leader in all project activities at the family level. He or she is the key contact person for Heifer and CCBRT. This becomes an authentic partnership between the families that creates its own synergy. For example, the family holds the responsibility to hand goats over to other families in the community. The aim of the project is to give families an opportunity to improve their income through production of goat’s milk. The families are also required to pass on the gift of the goat they have received by giving the first kid to another poverty-stricken family identified by the CRWs.

The aim of ‘passing on the gift’ is to teach the initial family that they too have the ability to help another family improve their quality of life. In addition, fertile goats can be sold for $100.00 per goat. Older female goats and surplus males can be slaughtered or sold for meat. Male goats may be used to fertilise a neighbour’s goats, thus continuing the cycle of passing on the gift and lifting more families out of poverty. Goat’s milk is rich in vitamin A and has proteins that are essential for the growth and development of CWDs. The family can sell surplus milk at more than three times the price of cow’s milk. Furthermore, goat’s milk can generate value-added products, such as cheese and yogurt. Manure from the goats, which is high in nitrogen, can be used to improve the soil, thus leading to increased crop yields. It can also be used to produce biogas (produced naturally from cow, goat and other animal manure) for cooking and lighting.

In addition, goats multiply quickly and can give birth twice a year, often producing twins or triplets. This increases the number of goats in a short period of time, leading to an increased number of beneficiary families. CCBRT-Moshi and Heifer International plan to invite an additional 100 beneficiaries to join the Pass on the Gift Project every year. In five years, approximately 300 families will benefit from 50 new kids per year.

## Results of the project

The results of this project in terms of poverty alleviation and hunger eradication have not yet been empirically verified. However, observational and anecdotal evidence suggests that families with CWDs have better quality of life through improved income generation and ultimately improved health through this synergistic partnership. Parents of CWDs have more than the usual expenses of raising a child. In addition to school fees, which average 100 000 TZS or $62.00 a year, families with CWDs often have to pay for doctor visits, surgery and hospitalisation costing over 1 500 000 TZS or $928.00 a year. CCBRT hopes that the outcome of this project will be to increase the number of CWDs receiving appropriate health care and attending school because their parents have the funds to spend on these necessities.

Occupational and physical therapists on the CCBRT staff as well as the CRW monitor control the services for income generation and the disabled children’s progress. Since this programme is still unfolding, CCBRT staff and administration continue to fine-tune the collaboration to ensure that families benefit from this synergy while the CBR model moves closer to CBR guidelines.

Through this project, mothers with CWDs are empowered financially to ensure the sustainable development of their families. The project aims to see a reduction in child mortality and the number of children born with a disability as a result of malnourished pregnant mothers and poor living conditions or lack of basic needs. Comprehensive Community Based Rehabilitation Tanzania and the Pass on the Gift Project are currently working on developing indicators to use as outcome measures to monitor the impact of this project on families and their children with disabilities.

### Project challenges, perceptions and the future

Like any other project, this partnership faces many challenges. At family level, the first challenge is to help families and caregivers to recognise the potential of their child with a disability, to encourage them to take the time to allow their child to engage in meaningful activities each day. Another challenge is to encourage families to alter their daily routines to include raising goats, and to change their livestock keeping practice to suit the needs of this new livestock that they have been given. Another challenge is for families to build the goat huts in a more timely and efficient manner. Families are building the goat huts very slowly. Although one might think that rain or the lack of access to raw materials or family sickness is slowing down the building process, there seems to be another reason. The problem stems from the fact that CCBRT staff and Heifer collaborators are trying to change the mindset of families. Families are asked not only to accept the process of building a goat hut, but also to accept the fact that, in most cases, the goat huts are made of higher quality materials than their own houses and are thus sturdier structures. Even the roofs of these goat huts are made of higher quality materials than their own houses – iron sheets, while the roofs of their homes are made of grasses or banana leaves.

Raising healthy goats is another challenge, as the goats are often infected with diseases that can lead to their death. One reason these diseases occur is because some goats are from a geographic region much different from their new area and their biological systems may have a difficult time adjusting to the different climate. Mating seasons are different depending on the local climate, too. Another challenge is families adapting to new goat-keeping practices versus the traditional way of keeping goats. Some deaths result from failure of veterinarians to give follow-up care and monitor progress. For the most part, educating families and continuously encouraging them can help the project overcome these challenges.

At an institutional level, another challenge is the bureaucracy and rampant corruption. For example, most of these veterinarians are employed by the government and seconded to the Heifer projects. It is difficult to control their activities and supervise their work because they have other government projects that they are supervising. Veterinarians have a heavy workload and having to oversee government projects and other existing Heifer projects makes it difficult for them to focus on this special project, although they are being paid for their participation and work. This challenge is not so easily addressed because they are government employees. This creates tension with the partnership, as the veterinarian plays an important role in creating synergy within this partnership.

## Conclusion

Through this project, the authors have come to an important realisation that has changed the way they see the issue of poverty and hunger. They believe that one’s life perspective and ultimately the condition of one’s spirit are key issues related to poverty and hunger. However, the challenges of hunger and poverty may be reduced and even overcome when an interdisciplinary, thoughtful, committed group of CBR professionals, an IG organisation, local government officials and families work together to create solutions that are centred on the client and the family. These solutions not only draw out the talents of all involved, including children with disabilities, but also lift up and build upon each person’s human capabilities in a synergy that is life-promoting, life-saving and life-giving for all.
